# **Metformin associates with higher myocardial perfusion reserve and survival in type 2 diabetes mellitus patients**

**DOI:** 10.1038/s41598-024-77280-2

**Published:** 2024-11-08

**Authors:** Noor Sharrack, Kristopher D. Knott, Gaurav S. Gulsin, Tushar Kotecha, Louise A. E. Brown, Jian L. Yeo, Aldostefano Porcari, Robert D. Adam, Sharmaine Thirunavukarasu, Amrit Chowdhary, Eylem Levelt, James C. Moon, Gerry P. McCann, Marianna Fontana, Peter Kellman, Theresa Munyombwe, Chris P. Gale, David L. Buckley, John P. Greenwood, Peter P. Swoboda, Sven Plein

**Affiliations:** 1 Department of Biomedical Imaging Science, Leeds Institute of Cardiovascular and Metabolic Medicine, University of Leeds, Leeds, LS2 9JT UK; 2 Barts Heart Centre, St Bartholomew’s Hospital, London, UK; 3 Institute of Cardiovascular Science, University College London, London, UK; 4 Department of Cardiovascular Sciences, Cardiovascular Biomedical Research Centre, University of Leicester and the NIHR Leicester, University Hospitals of Leicester NHS Trust, Glenfield Hospital, Leicester, UK; 5 Division of Medicine, National Amyloidosis Centre, University College London, Royal Free Campus, London, UK; 6 Centre for Diagnosis and Treatment of Cardiomyopathies, Cardiovascular Department, Azienda Sanitaria Universitaria Giuliano-Isontina (ASUGI), University of Trieste, Trieste, Italy; 7 Carol Davila University of Medicine and Pharmacy, Bucharest, Romania; 8 Department of Health and Human Services, National Heart, Lung, and Blood Institute, National Institutes of Health, Bethesda, MD USA; 9 Leeds Institute of Cardiovascular and Metabolic Medicine, University of Leeds, Leeds, UK; 10 Leeds Institute for Data Analytics, University of Leeds, Leeds, UK; 11 Department of Cardiology, Leeds Teaching Hosptislas NHS Trust, Leeds, UK

**Keywords:** Type 2 diabetes mellitus (T2DM), Myocardial perfusion reserve (MPR), Stress myocardial blood flow (MBF), Metformin, Endothelial dysfunction, Major adverse cardiovascular events (MACE), Cardiology, Diabetes

## Abstract

Metformin is an antihyperglycemic used to treat type 2 diabetes mellitus (T2DM). Patients with T2DM are at increased risk of cardiovascular disease. We explored the association between metformin use and cardiovascular magnetic resonance (CMR) derived stress myocardial blood flow (MBF), myocardial perfusion reserve (MPR) and major adverse cardiovascular events (MACE; all cause death, MI, stroke, heart failure hospitalisation and coronary revascularisation) in patients with T2DM. Multi-centre study of patients with T2DM, and healthy controls, underwent quantitative myocardial perfusion CMR using an artificial intelligence supported process. Multivariable regression analysis, and cox proportional hazard models of propensity score weighted patients quantified associations between metformin use, MBF, MPR, all cause death and MACE. Analysis included 572 patients with T2DM (68% prescribed metformin) with median follow-up 851 days (IQR 935 − 765). Metformin use was associated with an increase of MPR of 0.12 [0.08–0.40], *p* = 0.004. There were 82 MACE events (14.3%) including 25 (4.4%) deaths of which 16 were in those not prescribed metformin (8.7%), compared to 9 in patients prescribed metformin (2.3%): adjusted hazard ratio 0.24 (95% CI 0.08–0.70, *p* = 0.009). MACE events were similar between groups. This multicentre, inverse probability weighting propensity score analysis study showed that in patients with T2DM, metformin use is associated with higher MPR and improved all cause survival.

**Supplementary Information:**

The online version contains supplementary material available at 10.1038/s41598-024-77280-2.

## Introduction

Cardiovascular disease is the commonest cause of death in patients with Type 2 Diabetes Mellitus (T2DM)^1^. T2DM is associated with an increased risk of epicardial coronary artery disease (CAD), silent myocardial infarction (MI) and coronary microvascular dysfunction (CMD)^2–4^.

Metformin is the most widely used antihyperglycemic agent worldwide and is first-line in most recommendations for the treatment of patients with T2DM^5–8^. As well as its glucose lowering and weight loss benefits, it was also shown to be associated with a 39% lower risk of myocardial infarction compared to conventional therapy in the United Kingdom Prospective Diabetes (UKPDS) Study^9^. In a more recent study, metformin use was associated with a nearly 40% reduction in cardiovascular mortality when compared to sulfonylurea monotherapy^10^.

Metformin may improve endothelial function as well as reducing insulin resistance in patients with T2DM^11,12^. In non-diabetic women with chest pain and angiographically normal coronary arteries, metformin was shown to improve coronary microvascular function and decrease MI incidence^13^. In the retrospective Prevention of REStenosis with Tranilast and its Outcomes (PRESTO) trial, the use of metformin in diabetic patients undergoing coronary intervention decreased the risk of death and MI (relative risk reduction of 79%) compared to patients treated with a sulfonylurea or insulin^14^. Nevertheless, the mechanism of benefit of metformin beyond its glucose lowering effects is not clear and warrants further investigation, since meta-analyses of intensive versus normal glucose control have shown minimal benefit on cardiovascular outcomes^15^.

CMR allows simultaneous assessment of cardiac function, detection of MI, regional ischaemia suggesting epicardial CAD and, with quantitative perfusion analysis, absolute measures of myocardial blood flow (MBF) for the assessment of coronary microvascular dysfunction. Previous studies have shown that patients with T2DM have reduced global myocardial perfusion reserve (MPR) and stress MBF, independent of significant flow limiting epicardial CAD, suggestive of CMD^16,17^. To the best of our knowledge, to date, there are no published research studies that have used CMR to investigate the effects of metformin on MBF or MPR in humans, highlighting a clear lack of evidence in the field. We aimed to test the hypothesis that metformin use in patients with T2DM is associated with higher stress MBF and MPR and is associated with higher survival and reduced major adverse cardiovascular and cerbrovasular events (MACE; a composite of all-cause mortality, MI, stroke, heart failure hospitalisation and coronary revascularisation).

## Methods

This was a retrospective, multicentre, longitudinal cohort study in individuals with T2DM and a group of healthy volunteers who acted as a comparison group.

### Participants

Patients aged 18 years or older, with a diagnosis of T2DM, undergoing quantitative stress perfusion CMR, were recruited from four United Kingdom centres (St Bartholomew’s Hospital, Barts Heart Centre, London; Glenfield Hospital, University Hospitals of Leicester NHS Trust, Leicester; Leeds Teaching Hospitals NHS Foundation Trust, Leeds and the Royal Free Hospital, Royal Free London NHS Foundation Trust, London) between September 2016 and May 2021. Diagnosis of T2DM was based on a HbA1c > 48mmol/l or a known diagnosis of T2DM. Exclusion criteria included contraindications to adenosine, gadolinium-based contrast or MRI.

All participants had received quantitative myocardial perfusion stress CMR for clinical or research indications. Those with sub-optimal image quality, mistriggering or other artefacts were excluded.

### Controls

We recruited 52 age- and sex-matched, healthy controls with no past medical history of cardiac disease or major risk factors from two cardiac centres (Leeds Teaching Hospitals and Glenfield Hospital) between September 2016 and May 2021 to act as an external independent comparison to the two groups (prescribed metformin and not prescribed metformin). Exclusion criteria included a known past medical history of hypertension, hypercholesterolaemia, diabetes mellitus (DM), smoking, previous CAD or revascularisation or perfusion defect on stress CMR, contraindications to adenosine, gadolinium-based contrast or MRI and subsequent evidence of abnormal late gadolinium enhancement (LGE) on CMR.

### Clinical outcomes

Patient co-morbidities, medication history to include metformin, and clinical outcomes were collated from electronic patient records and the National Health Service (NHS) Spine Portal through deterministic linkage using each patient’s unique NHS identification number. Co-morbidities recorded included hypertension, dyslipidaemia, atrial fibrillation, CAD, coronary revascularisation (percutaneous coronary intervention (PCI) or coronary artery bypass graft (CABG) surgery, MI, stroke or transient ischaemic attack (TIA) and cancer. All patients had provided written, informed consent and had more than one year of follow-up data available.

Patients were then divided into two groups; those prescribed metformin and those not prescribed metformin at the time of CMR.

The joint primary outcomes were all-cause mortality and MACE (a composite of all-cause mortality, MI, stroke, heart failure hospitalisation and coronary revascularisation). Where patients had more than one MACE, the first event was used for analysis.

### Cardiovascular magnetic resonance imaging

CMR scans were performed on 1.5T (Magnetom Aera, Siemens Healthcare, Erlangen, Germany) or 3T (Prisma, Siemens Healthcare, Erlangen, Germany) scanners with a standard protocol consisting of localisers, short-axis and long-axis cine imaging, perfusion imaging and late gadolinium enhancement (LGE) imaging. 169 (30%) patients were scanned on 1.5T and 403 (70%) patients were scanned on 3T. All healthy controls were scanned on 3T. Perfusion imaging on 1.5T scanners used a steady state free precession (SSFP) pulse seqeunce and on 3T scanners a fast single-shot gradient echo (FLASH) pulse sequence. All participants were instructed to abstain from caffeine for 24 h before the study. For perfusion imaging, adenosine was infused for a minimum of 3 min, at a rate of 140 micrograms/kg/min and increased up to a maximum of 210 micrograms/kg/min if there was insufficient haemodynamic response (heart rate increase less than 10 bpm) or there was no symptomatic response, in line with standard clinical practice guidelines^18^. Images were acquired during free breathing over at least 60 dynamics to allow for any patients with poor ventricular function and consequent slow contrast uptake. A minimum interval duration of ten minutes was kept between stress and subsequent rest perfusion acquisitions. Participants’ blood pressures and heart rates were recorded at regular intervals during adenosine infusion. For each perfusion acquisition, an intravenous bolus of (0.05-0.75mmol/kg) gadobutrol (Gadovist, Leverkusen, Germany) or gadoterate meglumine (Dotarem, Guerbet, Paris, France) was administered at 4-5 ml/s. Perfusion mapping was performed in three short axis sections, using a dual sequence technique combining a low-resolution arterial input function acquisition and a higher resolution myocardial perfusion acquisition as previously described by Kellman et al.^19^.

### Image analysis

Image analysis was undertaken by experienced operators at the site core lab. Those with poor image quality were excluded. Measurement of cardiac volume parameters and the presence of LGE were made using cvi42 software (Circle Cardiovascular Imaging, Calgary, Canada). Left ventricular systolic and diastolic volume, ejection fraction, and the presence and distribution (ischaemic or non-ischaemic) of LGE were recorded. Non-ischaemic LGE refers to late gadolinium enhancement in a mid-myocardial or subepicardial pattern, as opposed to ischaemic LGE, which is subendocardial. Rest and stress perfusion maps were generated inline by an automatic artificial intelligence-supported process whereby myocardial blood flow is quantified for each pixel of the myocardium in ml/g/min. By averaging all pixel values in the 3 slices, global MBF and global MPR (ratio of stress to rest MBF) were derived^19^. Automatically derived perfusion maps were reviewed by experienced observers to exclude data affected by gating and motion correction artefact and partial voluming affects.

### Statistical analysis

Statistical analysis was performed using SPSS (IBM SPSS Statistics, version 27.0) and Stata (version 18.0). Normality was assessed through the Shaprio-Wilk test and variance was assessed by the Leven’s test for equality of variance. Continuous variables are presented as mean ± SD. Categorical variables are presented as absolute values and percentages. Means were compared using the appropriate test, student *t* test for continuous variables and chi-squared (χ^2^) test for categorical variables. A *P* value of < 0.05 was considered statistically significant. Multivariable linear regression analysis, adjusting for age, sex, left ventricular ejection fraction (LVEF), left ventricular mass (LV mass), left ventricular end diastolic volume (LVEDV), hypertension, evidence of previous PCI, CABG surgery, MI, body surface area (BSA), ethnicity as well as medications including calcium channel blockers (CCBs), nitrates, sodium glucose cotransporter 2 inhibitors (SGLT-2 inhibitors), glucagon-like peptide 1 (GLP-1) agonists and gliclazide was undertaken to seek association between metformin, stress MBF and MPR. Cox proportional hazard models adjusting for comorbidities and CMR parameters sought associations between stress MBF and MPR with all-cause mortality and MACE. For the all-cause mortality model, we adjusted for age and LVEF (only two predictors using the 10 events per parameter rule of thumb) and for the MACE model we adjusted for 8 parameters including age, LVEF, sex, LVEDV, LV mass, stress MBF, MPR and hypertension. To account for the lack of randomisation and non modifiable limitations of the study, inverse probability treatment weighting propensity score analysis applying cox proportional hazard models was employed. Kaplan–Meier curves represented event free survival and were estimated by the Nelson-Aalen method. Patient data was categorised depending on whether the patient was prescribed metformin at the time of the CMR scan.

## Results

A total of 572 patients with T2DM were included with a median follow-up of 851 days (IQR 935 − 765) days.

### Patient characteristics

Patient characteristics are shown in Table 1 and are characterised according to whether subjects were prescribed metformin at the time of the CMR.

**Table 1:** Patient demographics by metformin status.

	Metformin (*n* = 388)	No metformin (*n* = 184)	*P* value
Demographics			
Age (years)	64 ± 10	66 ± 11	0.057
Male sex	252 (65)	125 (68)	0.482
White	222 (57)	122 (66)	0.038*
Asian	125 (32)	40 (22)	0.010*
Black	20 (5)	10 (5)	0.888
Other	20 (5)	12 (7)	0.506
BSA (m^2^)	1.98 ± 0.2	1.97 ± 0.3	0.942
Previous PCI/CABG/MI	107 (28)	35 (19)	0.027*
Current smoker	83 (22)	46 (25)	< 0.001*
Former smoker	59 (15)	51 (28)	< 0.001*
Never smoked	245 (63)	87 (47)	< 0.001*
HTN	222 (58)	106 (58)	0.926
High cholesterol	241 (62)	98 (54)	0.044*
Previous stroke/TIA	16 (4)	18 (10)	0.008*
AF	39 (10)	39 (21)	< 0.001*
PVD	37 (10)	7 (4)	0.016*
HbA1C (mmol/mol)	60 ± 16	56 ± 15	0.022*
Cholesterol (mmol/l)	4.4 ± 1.4	4.8 ± 1.4	0.074

P value is considered significant at the < 0.05 and indicated by *. Continuous variables are presented as mean+/- SD. Dichotomous variables are presented as number (%). BSA- body surface area, PCI- percutaneous coronary intervention, CABG- coronary artery bypass graft, MI- myocardial infarction, HTN- hypertension, TIA- transient ischaemic attack, AF- atrial fibrillation, PVD- peripheral vascular disease.

Their mean age was 65 ± 10 years and their mean HbA1c was 59 ± 16 mmol/mol. 25% of all patients (*n* = 142) had known CAD with previous MI, PCI or CABG surgery. 68% of all patients (*n* = 388) were prescribed metformin. Metformin positive and metformin negative groups were well-matched for age and sex. Patients prescribed metformin were more likely to have had previous PCI, MI or CABG surgery (28% vs. 19%, *P* = 0.027) compared to those not prescribed metformin. They were also more likely to be of Asian ethnicity (32% vs. 22%, *p* = 0.01), suffer from hypercholesterolaemia (62% vs. 54%, *P* = 0.044) and have peripheral vascular disease (10% vs. 4%, *P* = 0.016). Patients who were prescribed metformin had higher mean HbA1c levels than those not prescribed metformin (60 ± 16 mmol/mol vs. 56 ± 15 mmol/mol, respectively, *P* = 0.022). However, patients not prescribed metformin had a higher prevelance of atrial fibrillation (21% vs. 10%, *P* < 0.001), previous stroke or transient ischaemic attack (10% vs. 4%, *P* = 0.008) and history of current smoking (25% vs. 22%, *p* < 0.001) compared to the non-metformin cohort.

Patients prescribed metformin were more likely to be on antiplatelet medication, statins, gliclazide, sodium glucose cotransporter 2 (SGLT-2) inhibitors, glucagon-like peptide 1 (GLP-1) agonists and calcium channel blockers (CCBs) (Table 2). However, patients not prescribed metformin, were more likely to be on mineralocorticoid receptor antagonists (MRAs), diuretics or anticoagulants.

**Table 2:** Patient medication by metformin status.

Medication	Metformin (*n* = 388)	No Metformin (*n* = 184)	*P* value
Aspirin/ other antiplatelet	254 (65)	89 (48)	< 0.001*
DOAC/ Warfarin	41 (11)	34 (18)	0.012*
Statin	332 (86)	122 (66)	< 0.001*
Beta blocker	209 (54)	114 (62)	0.086
ACE/ARB/Entresto	266 (69)	134 (72)	0.478
MRA	29 (8)	35 (19)	< 0.001*
Diuretics	87 (22)	68 (37)	< 0.001*
CCB	116 (30)	40 (22)	0.034*
Nitrates	54 (14)	21 (11)	0.382
Insulin	53 (14)	33 (18)	0.199
Gliclazide	94 (23)	17 (9)	< 0.001*
SGLT2	44 (11)	9 (5)	0.037*
GLP-1	61 (16)	15 (8)	0.011*
Gliptin	25 (6)	5 (3)	0.059

P value is considered significant at the < 0.05 and indicated by *. Dichotomous variables are presented as number (%). DOAC- Direct oral anticoagulant; ACE- Angiotensin converting enzyme inhibitor; ARB- Angiotensin receptor blocker; MRA- Mineralocorticoid receptor antagonist; CCB- Calcium channel blocker; SGLT2- Sodium glucose co-transporter 2 inhibitor; GLP-1-Glucagon like peptide agonist.

### Healthy control group

Subjects in the healthy control group were not taking any medication. The mean age was 64 ± 6 years and 65% of them were of male sex.

### CMR analysis

#### Patient group

169 (29.5%) of patients were scanned on 1.5T scanners and 403 (70.5%) were scanned on 3T scanners.

The mean ejection fraction of all diabetic patients was 57 ± 15%. 164 (29%) had evidence of ischaemic scar on LGE indicating previous MI. Mean global stress MBF in all patients was 1.70 ± 0.6 ml/g/min and mean MPR was 2.20 ± 0.90, which was significantly lower than in the healthy controls, (MBF 2.05 ± 0.4 ml/g/min, *p* < 0.0001 and average MPR was 3.40 ± 0.7, *p* < 0.0001).

Patients prescribed metformin had significantly higher ejection fractions (59 ± 14% vs. 53 ± 18%, *P* < 0.001), stress MBF (1.74 ± 0.6 ml/g/min vs. 1.62 ± 0.5 ml/g/min, *P* = 0.019), MPR (2.29 ± 0.9 vs. 1.96 ± 0.9, *P* < 0.001) as well as significantly lower EDV and LV mass (Table 3). There was no significant difference in the degree of scar burden between the two groups.

**Table 3:** CMR parameters by metformin status.

CMR Parameter	Metformin (*n* = 388)	No metformin (*n* = 184)	*P* value
LVEF (%)	59 ± 14	53 ± 18	< 0.001*
LVEDV (ml)	149 ± 50	174 ± 71	< 0.001*
LV mass (g)	117 ± 34	127 ± 39	0.002*
Global stress MBF (ml/g/min)	1.74 ± 0.6	1.62 ± 0.5	0.019*
Adjusted global stress MBF** (ml/g/min)	1.74 ± 0.03	1.67 ± 0.04	< 0.0001*
MPR	2.29 ± 0.9	1.96 ± 0.9	< 0.001*
Adjusted MPR**	2.27 ± 0.04	2.03 ± 0.07	< 0.0001*
LGE ischaemic	115 (30)	49 (27)	0.502
LGE non-ischaemic	89 (23)	50 (27)	0.502
No LGE	183 (47)	84 (46)	0.502
1.5T scanner	127 (33)	42 (23)	0.015*
3T scanner	261 (67)	142 (77)	0.015*

P value is considered significant at the < 0.05 and indicated by *. Continuous variables are presented as mean ± SD. Dichotomous variables are presented as number (%). ** Adjusted for age, sex, LVEF, LV mass, LVEDV, hypertension, evidence of previous PCI, CABG, MI, BSA, ethnicity, CCB, nitrates, SGLT2, GLP-1 and gliclazide. MBF- Myocardial blood flow; MPR-Myocardial perfusion reserve; LV- Left ventricle; LVEF- Left ventricular ejection fraction; LVEDV- Left ventricular end-diastolic volume; LGE-Late gadolinium enhancement, PCI- percutaneous coronary angioplasty, CABG- coronary artery bypass, MI- myocardial infarction, BSA- body surface area, CCB- Calcium channel blocker; SGLT2- Sodium glucose co-transporter 2 inhibitor; GLP-1-Glucagon like peptide agonist.

### Multivariable regression analysis

Multivariable linear regression was carried out to ascertain the extent to which metformin use is associated with stress MBF and MPR. The model was adjusted for factors known to be associated with differences in stress MBF and MPR including age, sex, LVEF, LV mass, LVEDV, hypertension, evidence of previous PCI, CABG surgery, MI, body surface area (BSA) and ethnicity as well as for medications including CCBs, nitrates, SGLT-2 inhibitors, GLP-1 agonists and gliclazide. Adjusted stress MBF and MPR values are reported in Table 3. For stress MBF, the regression coefficient for metformin had a value of 0.05 ([0.04–0.17], *P* = 0.21). This suggests that metformin use was associated with 0.05 ml/g/min higher stress MBF although this did not meet statistical significance. For MPR, the regression coefficient for metformin had a value of 0.12 ([0.08–0.40], *P* = 0.004). This suggests that the use of metformin is associated with 0.12 higher MPR, with statistical significance.

### Clinical outcomes

There were 82 first MACE (14.3%) including a total of 25 (4.4%) deaths, 15 (2.6%) MIs, 18 (3.1%) strokes, 20 (3.5%) heart failure hospitalisations and 43 (7.5%) episodes of coronary revascularisation.

Table 4 shows first MACE events by metformin status. Although there was no significant difference in first MACE rates between the two groups, there was a lower number of deaths in the metformin group (9 (2.3%) vs. 16 (8.7%), *P* < 0.001). There was also a significantly lower incidence of stroke in the metformin group (8 (2.1%) vs. 10 (5.4%), *P* = 0.031).

**Table 4:** MACE events by metformin status.

	Metformin (*n* = 388)	No Metformin (*n* = 184)	*P* value	All patients (*n* = 572)
MACE	53 (13.7)	29 (15.8)	0.503	82 (14.3)
Death	9 (2.3)	16 (8.7)	< 0.001*	25 (4.4)
MI	12 (3.1)	3 (1.6)	0.307	15 (2.6)
Stroke	8 (2.1)	10 (5.4)	0.031*	18 (3.1)
Heart failure hospitalisation	12 (3.1)	8 (4.3)	0.445	20 (3.5)
Coronary revascularisation	29 (7.5)	14 (7.6)	0.955	43 (7.5)

P value is considered significant at the 0.05 level and indicated by *. Data represented as number of events with percentage in brackets.

**Fig. 1:**
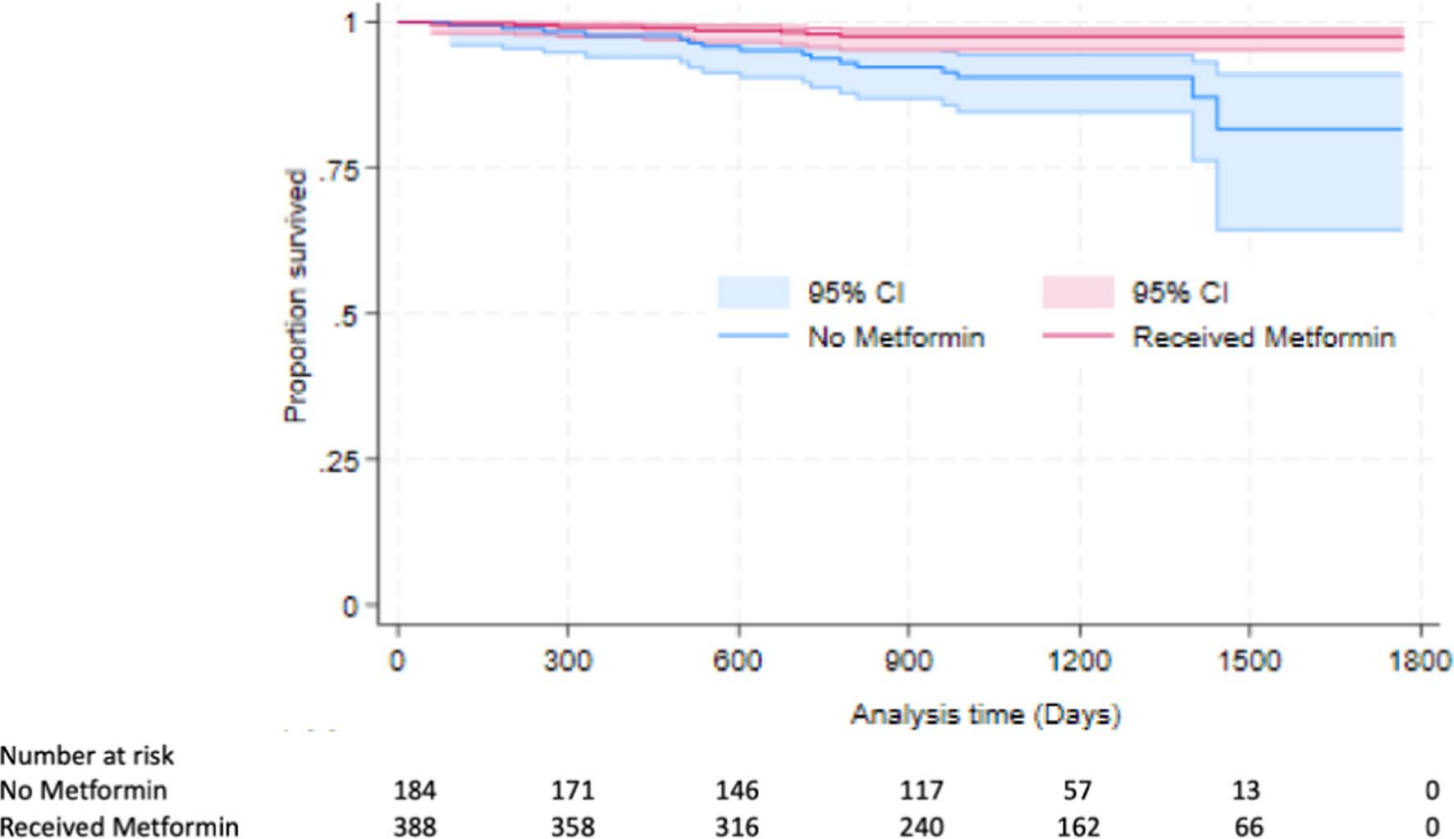
Kaplan–Meier survival curves and at risk table for all cause mortality per metformin status. P value < 0.001*. P value is considered significant at < 0.05.

Metformin was significantly associated with lower all-cause mortality (Fig. 1). After adjusting for age and LVEF, the adjusted hazard ratio for all-cause mortality was 0.29 ([0.12–0.73], *P* = 0.009 (Table 5)). However, metformin use was not associated with reduced first MACE rate after adjusting for age, LVEF, sex, LVEDV, LV mass, stress MBF, MPR and hypertension with an adjusted HR of 0.92 ([0.56–1.50], *P* = 0.73) (Table 5).

**Table 5:** Cox proportional hazard models.

	Death	MACE
Unadjusted		
Hazard ratio (Metformin)	0.22 [0.10–0.52], *p* < 0.001*	0.87 [0.55–1.37], *p* = 0.55
Adjusted		
Hazard ratio (Metformin)	0.29 [0.12–0.73], *p* = 0.009*	0.92 [0.56–1.50], *p* = 0.73

Death model was adjusted for age and LVEF. MACE model was adjusted for age, sex, LVEF, LVEDV, LV mass, stress MBF, MPR, HTN. Brackets represent confidence intervals. P value considered significant at < 0.05 and indicated by *.

LVEF- left ventricular ejection fraction, LVEDV- left ventricular end diastolic volume, MBF- Myocardial blood flow; MPR-Myocardial perfusion reserve; MACE- Major adverse cardiovascular event, HTN-hypertension.

### Inverse probability weighting propensity score analysis

To account for the lack of randomisation and non modifiable limitations of the study, inverse probability of treatment weighting propensity score analysis applying cox proportional hazard models was employed as described^20^. The variables included in estimating the propensity score consisted of: age, sex, LVEF, LVEDV, hypertension, evidence of previous PCI, CABG or MI, BSA and ethnicity as well as medications including CCBs, nitrates, SGLT-2 inhibitors, GLP-1 agonists and gliclazide. Balance for variables was checked using standardised mean difference and variance ratios (Table 6). The assumption was not violated and the covariates were balanced. Overlap assumption assessment was conducted (supplement, Fig. 1 ). After weighting and adjustment, cox proportional hazard modelling showed that metformin use was associated with a significant reduction in all cause mortality with a hazard ratio of 0.24, (95% CI 0.08–0.70), *P* = 0.009.

**Table 6:** Evaluating balance by assessing mean standardised differences and variance ratios.

Variable	Mean Standardised differences		Variance Ratio	
	Raw data	Weighted data	Raw data	Weighted data
Age	-0.150	-0.006	0.836	0.942
Sex (male)	-0.076	-0.437	1.051	1.033
EF	0.447	-0.076	0.600	0.909
LVEDV	-0.418	-0.631	0.470	0.860
HTN	0.003	0.026	0.996	0.993
Previous PCI/CABG/MI	0.301	0.018	1.706	1.028
BSA	0.025	0.001	0.600	0.735
White Caucasian	-0.180	-0.003	1.082	1.001
Nitrates	0.061	0.004	1.142	1.009
Calcium channel blocker	0.172	0.005	1.204	1.005
SGLT-2	0.112	0.019	2.324	1.143
GLP1	0.209	-0.111	1.663	0.807
Gliclazide	0.397	-0.042	2.120	0.941

A balanced variable has a mean standardised difference of zero and variance ratio of one in the weighted data. EF-ejection fraction; LVEDV- left ventricular end diastolic volume, HTN-hypertension, PCI- percutaneous coronary intervention; CABG- coronary artery bypass grafting; BSA- body surface area; SGLT-2- Sodium glucose co-transporter 2 inhibitor; GLP-1 agonist-Glucagon like peptide agonist.

## Discussion

**Fig. 2:**
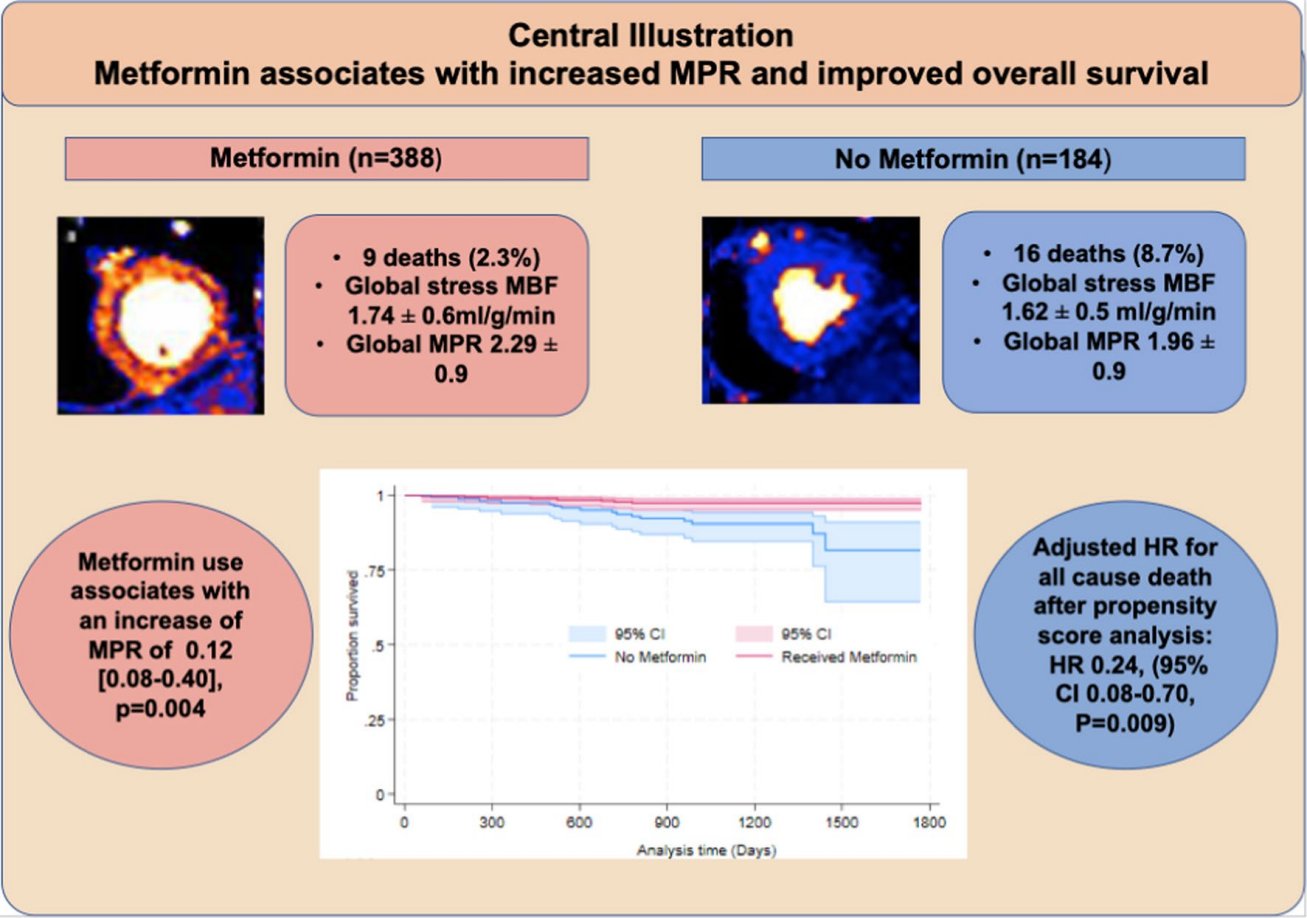
This retrospective, multicentre, longitudinal cohort study showed that in patients with T2DM, metformin use is associated with higher MPR on quantitative perfusion cardiovascular magnetic resonance imaging and improved all cause survival.

We sought to test the hypothesis that metformin use in patients with T2DM is associated with higher coronary microvascular function, higher survival and lower MACE rates. The key findings in this study are that metformin use is associated with (1) higher MPR as a marker of coronary microvascular function and (2) lower all-cause mortality after adjusting for important parameters (Fig. 2).

### Association between metformin and MPR

To the authors knowledge, this is the first clinical study that has demonstrated an association between metformin use and higher coronary microvascular function in patients with T2DM. Obesity, insulin resistance, and endothelial dysfunction closely co-exist throughout the natural history of T2DM^21^. Endothelial dysfunction is considered an important event in the development of microvascular complications in diabetes and is well recognised in patients with T2DM. Insulin resistance itself may be central to the pathogenesis of endothelial dysfunction. Patients with T2DM have been shown to have reduced MPR as manifestations of CMD caused by endothelial dysfunction^16,17^. Metformin has been shown to improve endothelial function^22^. A previous study conducted in 16 asymptomatic patients recently diagnosed with T2DM using Positron Emission Tomography (PET), looked at the effect of metformin treatment alone or with a combination of glimepiride/metformin on coronary endothelial function^23^. It showed that at baseline, the response of T2DM patients to cold pressor test was reduced compared to controls, demonstrating endothelial dysfunction. Following treatment with a combination of metformin/glimepiride, myocardial flow reserve (MFR) and response to cold pressor testing MBF (% delta MBF) improved. Another study conducted in patients with T2DM showed an improvement in endothelium-dependent vasodilatation after administering metformin, assessed through forearm plethysmography. This change was also accompanied by a reduction of insulin resistance^11^. A study in rats who were fed a high fat diet showed an improvement in MPR after administering a 4-week treatment of metformin^24^. This study also showed that metformin treatment led to a robust adenosine-induced increase in myocardial perfusion (+ 81%). The authors postulated that metformin improved cardiac perfusion by restoring balance between vasoconstrictor and vasodilator effects of modest insulin resistance. This study also looked at the effects of sulodexide treatment in rats. Sulodexide is a glycocalyx stabliser and has been shown to improve functions of the glycocalyx in diet-induced obese rats^25^. Glycocalyx is a proteoglycan-rich hydrogel that separates the blood from the endothelium, is important in maintaining integrity of the endothelium and has been shown to be disrupted in patients with T2DM^25^. This study showed that sulodexide treatment led to a + 37% adenosine-induced increase in myocardial perfusion. There has been suggestion that enhanced function of the glycolalyx may alleviate endothelial dysfunction and thereby improve myocardial perfusion. We postulate that an improvement in endothelial function through the use of metformin in patients with T2DM may lead to higher MPR. We speculate that this effect was not seen with stress MBF on multivariable regression analysis since MPR is a better measure of endothelial dysfunction^26^.

### Metformin and improved survival

Our data supports previous reports that metformin is associated with higher overall survival. Despite having worse cardiovascular profiles, patients on metformin had higher ejection fractions compared to patients not prescribed metformin. We speculate that this may be secondary to more intense risk factor modification and drug treatment effects, for example with SGLT-2 inhibitors and GLP-1 agonists, although we cannot establish the cause from this study alone. The United Kingdom Prospective Diabetes Study (UKPDS) showed that metformin use reduced diabetes-related mortality by 42% (*P* = 0.017), all-cause mortality by 36% (*P* = 0.011), MI by 39% (*P* = 0.01) and any diabetes related endpoint by 32% (*P* = 0.002)^9^. In a recent systematic review, looking at 40 studies, metformin was shown to reduce cardiovascular mortality, all-cause mortality and cardiovascular events in CAD patients^27^. However, for MI and CAD patients without T2DM, metformin had no effect of reducing the incidence of cardiovascular events. In our study, metformin was not associated with an improvement in MACE rate. This is not surprising since patients prescribed metformin had higher cardiovascular risk compared to patients not prescribed metformin. In patients prescribed metformin there was also an increased prevalence of peripheral vascular disease and hypercholesterolaemia. Supporting our data findings, a meta analysis that included 33 studies and 61,704 patients revealed that metformin use reduces all-cause mortality but with no reduction in the incidence of MI, angina or stroke^28^. Recently, additional benefits of metformin have been reported including potential effects on cancer^29,30^, cardiovascular disease^13^, liver disease^31^, obesity^32^, neurodegenerative disease^33^ and renal disease^34^. Extended release metformin has also been shown to improve cognitive function in frail old women with diabetes and hypertension and to mitigate fraility effects in pre-diabetes^35,36^. The association between metformin use and higher survival is unclear; further research needs to be undertaken in this field.

### Improving mitochondrial function

T2DM is a metabolic disorder characterised by mitochondrial dysfunction and oxidative stress^37^. By increasing nitric oxide (NO) bioavailability, limiting interstitial fibrosis, reducing the deposition of advanced glycation end products (AGEs), and inhibiting myocardial cell apoptosis, metformin has been shown to reduce cardiac remodelling and hypertrophy, thereby preserving left ventricular systolic and diastolic function^38,39^. Metformin has also been shown to exert cardioprotective effects against ischemia-reperfusion by improving mitochondrial function through the activation of a cell signalling pathway AMPK (AMP-activated protein kinase) and its downstream signalling molecule PPARα (peroxisome proliferator-activated receptor alpha)^40^. Metformin has also been shown to affect lysosomal AMPK pathways through stimulation of presenilin enhancer 2 (PEN2)^41^. Although we did not directly measure mitochondrial function in our study, these mechanisms may explain why patients taking metformin had higher ejection fractions, lower LVEDV and LV mass.

## Limitations

Several limitations should be acknowledged in this study. First, the study carries all the inherent limitations of an observational study and since subjects were non-randomised, the associations between metformin and higher survival and MPR found cannot imply causation and may be secondary to one or more confounders. One should also consider the potential for bias in the estimated coefficients due to the relatively small number of events and large number of co-variates. It is also likely that the two groups were profoundly different with regards to patient characteristics. For example, sicker patients may not have been prescribed metformin, leading to selection and treatment bias although this is unlikely to be the case in our cohort, since the patients prescribed metformin had more cardiovascular morbidity (increased prevalence of MI, PCI and CABG surgery, peripheral vascular disease and hypercholesterolaemia). It may also be plausible that patients prescribed metformin were better managed for conventional cardiovascular risk factors, due to the fact these patients tend to be higher risk patients. Furthermore, since events were documented using electronic patients records from different hospitals, there is a chance that events may have been missed. Patients on metformin in the current study were more likely to be on other medications such as gliclazides, nitrates, SGLT-2 inhibitors, GLP-1 agonists, and CCBs. Therefore, the higher MPR observed may have been mediated by one or more combination drug effects, and although we have adjusted for these in our multivariable linear regression analysis models and with further inverse probability treatment weighting propensity score analysis and there is no evidence in the literature that these medications affect MPR, it is not possible to conclude that metformin was independently associated with higher MPR and lower mortality with confidence. The effects of selection bias, treatment bias and other sources of bias detailed above may be responsible for imprecision in the authors conclusions. Furthermore, information on the duration of diabetes and the length of treatment with metformin was not collected in all patients. Therefore, our results must be considered hypothesis-generating and an indication that further research in this area, preferably in the form of a double blind, randomised prospective placebo-controlled clinical trial, would be able to address some of the points above.

## Conclusion

In patients with T2DM, we show that metformin use is associated with higher MPR, as a marker of coronary microvascular function, and is associated with higher overall survival. Further research in this area is important to support these preliminary findings.

## Electronic supplementary material

Below is the link to the electronic supplementary material.

Supplementary Material 1

## Data Availability

The datasets used and analysed during the current study are available from the corresponding author on reasonable request.

## Abbreviations

CAD: Coronary artery disease
CMD: Coronary microvascular dysfunction
CMR: Cardiovascular magnetic resonance
T2DM: Type 2 diabetes mellitus
LV: Left ventricle
LVEF: Left ventricular ejection fraction
MI: Myocardial infarction
MACE: Major adverse cardiovascular events
MBF: Myocardial blood flow
MPR: Myocardial perfusion reserve
