# Supplementary material for: Metformin associates with higher myocardial perfusion reserve and survival in type 2 diabetes mellitus patients
Source: Sci Rep. 2024 Nov 8;14:27280. doi: 10.1038/s41598-024-77280-2 (PMC11549305; doi:10.1038/s41598-024-77280-2)

**Assessing the overlap assumption**

*Figure S1: The analysis included participants that had equal chance of receiving treatment with most of them having a propensity score within the range 0.1 and 0.9.*


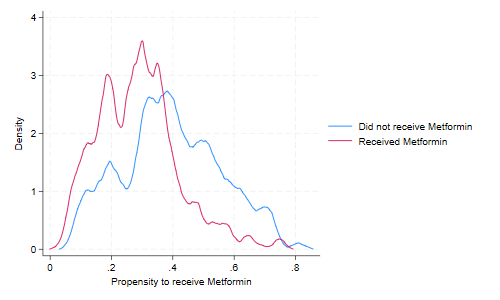

Supplement: Supplementary file 1 — Supplementary Material 1 [file 41598_2024_77280_MOESM1_ESM.docx]
